# A Formal Optimization-Oriented Design Framework for Predictive Extrusion-Based 3D Bioprinting

**DOI:** 10.3390/biomimetics11030165

**Published:** 2026-03-01

**Authors:** Antreas Kantaros, Theodore Ganetsos, Michail Papoutsidakis

**Affiliations:** Department of Industrial Design and Production Engineering, University of West Attica, 12244 Athens, Greece

**Keywords:** 3D bioprinting, biofabrication, tissue engineering, applied mathematical modeling, predictive design, process–structure–function relationships, constrained optimization, scaffold architecture, reproducibility and scalability, computational bioengineering

## Abstract

Extrusion-based three-dimensional (3D) bioprinting has enabled the fabrication of complex, cell-laden constructs; however, process parameter selection remains largely empirical and system-specific. As biofabrication workflows scale in complexity and translational ambition, trial-and-error optimization increasingly limits reproducibility, transferability, and informed decision-making. In this work, a formal, optimization-oriented design framework is proposed to structure extrusion-based bioprinting as a constrained, multivariable design problem. Rather than introducing a system-specific predictive model, the framework organizes process parameters, material descriptors, scaffold architecture, and biological feasibility into a unified formulation based on objective functions and admissible constraints. Symbolic coupling relationships are employed to make parameter dependencies, trade-offs, and constraint interactions explicit without imposing restrictive assumptions on material behavior or biological response. A demonstrative computational case study is presented to illustrate how qualitative predictive reasoning emerges through constraint-driven design space analysis and multi-objective considerations. The framework reveals how feasible operating regions are shaped by competing biological, mechanical, and manufacturing limitations, emphasizing robustness-aware parameter selection over isolated optimization. The proposed approach is intended as a transferable methodological foundation that supports structured reasoning, experimental planning, and future integration with numerical models, data-driven tools, and closed-loop biofabrication systems.

## 1. Introduction

Three-dimensional (3D) bioprinting has emerged as a transformative biofabrication technology, enabling the controlled spatial deposition of biomaterials, living cells, and bioactive components for the creation of tissue-engineered constructs with increasing structural and biological complexity [1]. Over the past decade, advances in printable biomaterials, extrusion and jetting systems, and cell-compatible processing conditions have significantly expanded the scope of bioprinting applications, ranging from in vitro tissue models and organoids to implantable therapeutic platforms [2]. As a result, 3D bioprinting is increasingly positioned as a key enabling technology for regenerative medicine, personalized therapies, and advanced biomedical research [3,4]. In this study, the focus is placed specifically on extrusion-based 3D bioprinting, which remains the most widely adopted biofabrication modality due to its material versatility, scalability, and compatibility with cell-laden bioinks.

Despite this rapid technological evolution, the practical realization of robust and clinically relevant bioprinted constructs remains strongly dependent on empirical parameter selection and iterative trial-and-error optimization [5]. Printing speed, extrusion pressure, nozzle geometry, material rheology, and crosslinking conditions are often adjusted heuristically, guided by prior experience or isolated experimental observations. While this empirical approach has enabled important proof-of-concept demonstrations, it presents fundamental limitations when bioprinting workflows are scaled toward reproducibility, standardization, and translation beyond laboratory settings [6].

One of the most persistent challenges concerns reproducibility [7]. Small variations in process parameters or material properties can lead to substantial differences in filament morphology, scaffold fidelity, and cellular response, complicating cross-study comparison and protocol transfer. Scalability presents an additional barrier, as parameter sets optimized for small constructs or single laboratories frequently fail to generalize to larger geometries, higher throughput systems, or alternative biofabrication platforms. Moreover, the predictability of biological outcomes remains limited, as the relationship between fabrication parameters, scaffold architecture, and subsequent cell viability, nutrient transport, and tissue maturation is rarely quantified in a unified and systematic manner [8].

Despite extensive research on extrusion-based bioprinting, current modeling and optimization approaches remain fragmented, often addressing individual aspects such as flow behavior, filament formation, or biological response in isolation. Moreover, parameter selection is still largely driven by empirical tuning, limiting reproducibility, transferability across systems, and predictive reasoning at the process design level. There is therefore a lack of unified methodological frameworks that explicitly integrate process parameters, material behavior, structural outcomes, and biological constraints within a single, optimization-oriented formulation for extrusion-based bioprinting.

While extrusion-based bioprinting shares mechanical similarities with conventional extrusion-based 3D printing, the presence of living cells fundamentally alters the nature of the optimization and control problem. In bioprinting, admissible process parameters are not determined solely by geometric accuracy or material deposition stability, but are additionally bounded by biological constraints such as shear-induced cell damage, nutrient transport limitations, and post-fabrication tissue viability. As a result, optimization objectives that are standard in conventional extrusion printing—such as maximizing throughput or dimensional fidelity—must be reformulated within a constrained, multi-objective design space dominated by biological feasibility. The novelty of the present framework lies not in redefining extrusion physics, but in explicitly restructuring extrusion-based fabrication as a biology-limited optimization problem, where predictive reasoning is guided by constraint interactions rather than purely mechanical performance.

These challenges highlight a critical gap in current biofabrication practice: the absence of predictive, quantitative methodologies capable of formally linking bioprinting process parameters to structural and biological performance metrics [9]. Without such methodologies, optimization remains local and system-specific, and biofabrication workflows struggle to achieve the reliability required for clinical translation and industrial adoption. Addressing this gap requires moving beyond descriptive experimentation toward frameworks that can support rational design, sensitivity analysis, and informed trade-off evaluation [10].

Applied mathematics offers a powerful yet underutilized enabling layer for addressing these limitations. Mathematical modeling, optimization, and control provide formal tools for representing complex process–structure–function relationships, exploring high-dimensional design spaces, and quantifying uncertainty and robustness [11]. When integrated into biofabrication workflows, applied mathematical frameworks can support predictive parameter selection, guide scaffold design under biological and manufacturing constraints, and establish a foundation for reproducible and scalable bioprinting strategies. Importantly, such frameworks do not replace experimental investigation but complement it by structuring decision-making and reducing reliance on exhaustive empirical tuning [12].

Against this background, the objective of this work is to propose a general applied mathematical framework for predictive extrusion-based 3D bioprinting in which process parameters, material properties, scaffold architecture, and biological performance indicators are treated within a unified formulation. Rather than introducing a system-specific model or validated simulation, the study aims to (i) structure extrusion-based bioprinting as a constrained, multi-objective design problem, (ii) clarify process–structure–function couplings relevant to biological fabrication, and (iii) demonstrate how predictive reasoning and robustness considerations can be supported at a methodological level. The framework is intended to serve as a transferable foundation for subsequent experimental, numerical, or data-driven implementations.

## 2. Motivation and Background

The rapid expansion of 3D bioprinting technologies has been accompanied by a diversity of fabrication strategies, materials, and application-driven objectives, resulting in biofabrication workflows that are often highly customized and system-specific. While this flexibility has accelerated innovation, it has also led to fragmented design practices that rely heavily on empirical knowledge and localized optimization [12]. As bioprinting systems move toward increased complexity, scale, and translational relevance, the limitations of these paradigms become increasingly evident. In particular, the absence of unifying methodological principles hinders the systematic comparison, transferability, and predictive control of biofabrication processes [13]. This section examines prevailing biofabrication approaches, identifies the structural limitations of empirical and isolated modeling strategies, and motivates the need for a unified applied mathematical methodology capable of supporting predictive, quantitative, and digitally integrated bioprinting workflows.

### 2.1. Current Biofabrication Strategies

Contemporary biofabrication workflows are predominantly shaped by empirical optimization strategies in which printing parameters are iteratively adjusted to achieve acceptable construct fidelity and biological compatibility. Variables such as extrusion pressure, printing speed, nozzle geometry, material concentration, and crosslinking conditions are typically tuned through sequential experimentation, often guided by practitioner experience or platform-specific heuristics [14]. While this approach has enabled rapid prototyping and system familiarization, it inherently treats each parameter in relative isolation and offers limited insight into their coupled effects on construct performance.

This parameter-by-parameter adjustment paradigm is further reinforced by the diversity of bioprinting platforms and biomaterial systems, which discourages the development of generalized design rules. As a result, optimization strategies are frequently confined to narrow operational windows and remain tightly coupled to specific printers, materials, or laboratory environments [15]. Even when successful outcomes are achieved, the underlying rationale for parameter selection is rarely formalized, constraining reproducibility and hindering systematic refinement.

Consequently, generalization across systems remains limited. Protocols optimized for one biofabrication setup often exhibit reduced performance or outright failure when transferred to alternative configurations, scales, or application contexts. This lack of transferability underscores a structural weakness in prevailing paradigms, where empirical success does not readily translate into predictive capability.

### 2.2. Limitations of Empirical and Isolated Modeling Approaches

In response to the shortcomings of purely empirical optimization, modeling and simulation tools have been increasingly adopted to analyze specific aspects of the bioprinting process. Computational fluid dynamics, mechanical simulations, and transport models have provided valuable insights into nozzle flow behavior, filament formation, scaffold mechanics, and nutrient diffusion [16,17]. However, these efforts are typically implemented in a fragmented manner, focusing on isolated subsystems rather than the biofabrication workflow as an integrated whole.

A central limitation of such isolated modeling approaches lies in the weak coupling between fabrication physics and biological performance. Mechanical or flow-based models may accurately describe process conditions, yet remain disconnected from biological constraints such as cell viability thresholds, shear sensitivity, or long-term tissue maturation. Conversely, biological assessments often lack a quantitative link to the process parameters that govern construct formation. This separation limits the capacity of models to inform holistic design decisions.

Moreover, the transferability of isolated simulations to clinically relevant scales is often limited. Models calibrated for simplified geometries or small constructs may not extrapolate reliably to larger volumes, complex architectures, or multi-material systems [18]. Without a unifying framework that explicitly accounts for parameter interactions, constraints, and trade-offs, both empirical and isolated modeling approaches struggle to support robust, scalable biofabrication strategies.

### 2.3. Need for a Unified Mathematical Methodology

The limitations of current examples point to the need for a unified methodological approach capable of integrating process parameters, scaffold architecture, and biological performance within a single predictive framework. Such an approach must move beyond descriptive experimentation and fragmented simulation toward formal representations that enable quantitative reasoning, systematic optimization, and informed decision-making.

Applied mathematics provides the conceptual and computational tools necessary to support this transition. By formulating biofabrication workflows in terms of defined variables, governing relationships, objective functions, and constraints, mathematical methodologies enable predictive design and sensitivity analysis across high-dimensional parameter spaces. This formalization allows trade-offs between competing objectives—such as print fidelity, biological viability, and manufacturing efficiency—to be explicitly identified and managed.

Importantly, a unified mathematical methodology also facilitates digital and computational integration. Model-based workflows can be coupled with simulation environments, optimization routines, and control strategies, forming the foundation for reproducible and scalable biofabrication systems. Within this context, applied mathematics serves not as an abstract layer, but as an enabling methodology that structures complexity and supports the evolution of biofabrication from empirical practice toward predictive, intelligent design. To synthesize the limitations of prevailing biofabrication paradigms and to clarify the methodological requirements for predictive 3D bioprinting, the key characteristics of current approaches are summarized and contrasted in Table 1.

## 3. Mathematical Framework for 3D Bioprinting

The complexity of extrusion-based 3D bioprinting arises from the coupled interaction of fabrication parameters, material response, scaffold architecture, and biological feasibility, all operating across multiple spatial and temporal scales. In practice, these interactions are rarely addressed in a unified manner; instead, parameter selection is typically performed through sequential empirical adjustment. The aim of the present framework is not to introduce a closed-form predictive model or to derive system-specific governing equations, but rather to formally structure the bioprinting process as a constrained design and optimization problem.

Within this context, the term predictive is used to denote constraint-driven reasoning—that is, the ability to anticipate admissible and non-admissible regions of the process design space prior to fabrication, rather than to compute quantitative biological outcomes [19]. The framework provides a formal representation of parameter interdependencies, objective trade-offs, and feasibility limits, enabling structured exploration of design decisions without reliance on trial-and-error experimentation.

Importantly, all relationships introduced in this section are expressed at a symbolic and phenomenological level. This abstraction is intentional: explicit functional forms for flow behavior, filament stability, or biological response depend strongly on material composition, printer architecture, and cell type, and therefore cannot be defined generically without introducing restrictive assumptions [20]. By maintaining symbolic representations, the framework remains transferable across biofabrication systems while preserving interpretability and methodological clarity.

### 3.1. Definition of the Biofabrication Design Space

The extrusion-based bioprinting process is formalized as a multivariable design space composed of controllable fabrication parameters, material descriptors, and resulting biological performance indicators. Process parameters are represented by a vector of decision variables,

p={P,u,d,T},

where P denotes the extrusion pressure, u the printing speed, d the nozzle diameter, and T the processing temperature. These variables define the operational degrees of freedom available during fabrication and collectively determine material transport and deposition behavior.

Material behavior is characterized through a set of effective descriptors,

m={n,k}

where n represents the effective viscosity of the bioink and k denotes parameters associated with solidification or crosslinking kinetics. These descriptors capture the dominant material response relevant to extrusion stability and shape retention without prescribing specific rheological laws.

Biological feasibility is represented through outcome indicators,

$$b=\left\{V,D,\;T_{crit}\right\}$$

where $V_{C}$ denotes cell viability proxies, D represents effective nutrient transport capacity, and $T_{crit}$ corresponds to admissible shear exposure thresholds. These quantities are not directly controlled but emerge as consequences of the interaction between process parameters, material response, and scaffold architecture.

Collectively, the framework defines a formal mapping,

(p,m)→b,

which encapsulates the process–structure–function logic of extrusion-based bioprinting. This mapping does not imply explicit analytical solvability; rather, it establishes a consistent representation through which feasibility, trade-offs, and constraint interactions can be systematically examined.

### 3.2. Formal Representations of Fabrication–Biology Couplings

While the present demonstrative case study incorporates biological performance primarily through proxy constraints—such as shear stress exposure during extrusion and nutrient transport within the printed construct—the proposed framework is inherently compatible with more detailed and dynamic biological representations. Time-dependent descriptions of cell activity, proliferation, and differentiation may be introduced through phenomenological evolution relations defined over the scaffold domain. Such representations allow biological performance indicators to evolve post-fabrication, linking early-stage fabrication conditions to longer-term tissue development without altering the formal structure of the framework.

Extrusion-based biofabrication involves a combination of physical and biological phenomena that unfold during material deposition and continue throughout post-printing maturation [21]. To formally organize these phenomena within a unified design framework, the present methodology adopts continuum-inspired representations that provide generality and interpretability while avoiding system-specific parameterization. These representations are **not introduced as solvable governing equations**, but rather as a consistent modeling language that clarifies how process parameters, material response, and biological feasibility are structurally coupled across diverse bioprinting contexts.

During fabrication, material transport and filament formation may be conceptually described using continuum flow representations, in which the bioink is treated as a viscoelastic or generalized Newtonian medium. At this level, relationships between process variables and material response are expressed symbolically, motivated by balance considerations and constitutive behavior, without pursuing analytical or numerical solution. This abstraction justifies the inclusion of parameters such as extrusion pressure, printing speed, and nozzle geometry within the design space and supports the formulation of biologically motivated constraints related to shear exposure and deposition stability.

Material behavior within the framework is represented through effective descriptors, including viscosity and crosslinking-related parameters, in order to preserve generality and analytical transparency. It is acknowledged that many bioinks exhibit more complex rheological responses, such as shear-thinning, thixotropy, or viscoelasticity. Constitutive formulations, including Herschel–Bulkley-type relations or linear viscoelastic descriptions, may be incorporated into the framework by refining these descriptors, without modifying its overall structure. Such extensions are viewed as system-specific implementations rather than requirements of the present methodological formulation.

Following deposition, transport phenomena within the printed construct play a central role in biological feasibility and longer-term function [22,23]. Nutrient diffusion, waste removal, and oxygen transport can be formally represented through diffusion–reaction-type descriptions defined over the scaffold domain, with effective transport parameters reflecting porosity and architecture induced by the fabrication process. These representations enable qualitative reasoning about spatial heterogeneity and support the inclusion of transport-related constraints within the design space, rather than providing quantitative prediction of biological outcomes.

Mechanical behavior of the printed construct is similarly accommodated through continuum-inspired representations. The scaffold may be treated as a deformable structure whose effective stiffness and integrity depend on material properties, crosslinking behavior, and deposition geometry. Time-dependent representations may account for post-printing solidification, swelling, or degradation processes that influence mechanical stability and long-term functionality. At this stage, such descriptions serve to identify relevant dependencies and constraint interactions rather than to compute mechanical response quantitatively.

Biological maturation introduces an explicit temporal dimension to the framework. Cell proliferation, extracellular matrix deposition, and tissue remodeling occur over timescales extending beyond fabrication and may be represented through phenomenological evolution relations. These time-dependent representations reinforce the distinction between immediate fabrication outcomes and longer-term biological function, underscoring the need for design frameworks that extend beyond static, geometry-centered assessments.

**Figure 1:**
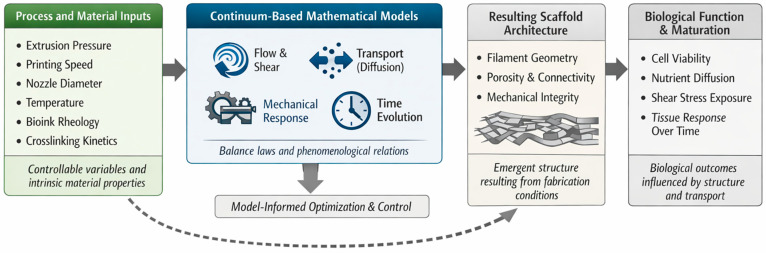
Conceptual schematic of the applied mathematical framework for 3D bioprinting, illustrating how continuum-based governing descriptions link process and material inputs to scaffold architecture and biological performance.

By employing continuum-inspired descriptions at a **symbolic and formal level**, the proposed framework establishes a coherent representation of flow, transport, mechanics, and biological evolution in extrusion-based 3D bioprinting. This approach prioritizes interpretability, transferability, and structured reasoning over exhaustive physical detail, creating a foundation upon which coupled process–structure–function relationships can be systematically explored in subsequent sections. A conceptual overview of these formal representations and their role in linking process inputs to structural and biological considerations is illustrated in Figure 1.

The coupling relationships introduced within the framework are intentionally expressed symbolically to emphasize dependency structure and constraint interaction rather than system-specific parameterization. Explicit functional forms for shear stress, shape fidelity, or biological response depend strongly on material composition, printer architecture, and cell type, and therefore cannot be defined generically without introducing restrictive assumptions. By maintaining symbolic representations, the framework makes the existence, directionality, and constraint-driven interaction between variables explicit, while allowing quantitative formulations to be incorporated in future, application-specific implementations.

### 3.3. Coupling Fabrication Parameters with Biological Function

A defining characteristic of biofabrication is the indirect and multistage relationship between controllable fabrication parameters and biological function [24]. Process variables do not influence biological performance directly; instead, their effects are mediated through the scaffold architecture and the transport and mechanical environments that emerge during and after fabrication [25]. Capturing this chain of dependencies is essential for **structured reasoning in bioprinting workflows** and represents a central challenge addressed by the proposed design framework.

At the first level, fabrication parameters determine the structural features of the printed construct, establishing a **process–structure relationship**. Extrusion pressure, printing speed, and nozzle diameter influence filament geometry, pore size, and interconnectivity, while material rheology and crosslinking kinetics govern shape retention and mechanical integrity [26]. These structural characteristics define the physical environment in which cells reside, constraining mass transport, mechanical loading, and spatial organization. Within the framework illustrated in Figure 1, scaffold architecture emerges as an intermediate—and critical—outcome that mediates between controllable process inputs and biological feasibility.

At the second level, scaffold architecture conditions biological response through a **structure–function relationship**. Porosity and connectivity influence nutrient diffusion and waste removal, while filament dimensions and effective stiffness affect cell attachment, proliferation, and mechanotransduction [27]. In addition, shear exposure during fabrication imposes viability-related thresholds that delimit admissible regions of the process design space. These biological responses span multiple spatial and temporal scales and often evolve as maturation and remodeling processes unfold, further reinforcing the indirect nature of process–biology coupling.

The interaction between process parameters, structure, and biological function is further complicated by nonlinear dependencies and competing objectives. For example, increasing printing speed may improve manufacturing efficiency while simultaneously increasing shear exposure, whereas enhanced crosslinking may improve mechanical stability at the expense of nutrient transport. In the absence of a formal framework, such trade-offs are typically resolved empirically and locally. In contrast, a **formal optimization-oriented formulation** allows these couplings to be expressed explicitly at a structural level, supporting systematic exploration of feasible design regions and transparent assessment of competing constraints without requiring system-specific calibration.

Importantly, the coupling challenges identified here are not merely technical but methodological. They arise from the lack of unified representations capable of integrating fabrication physics and biological considerations within a consistent design logic. By structuring these relationships within a formal framework, the biofabrication problem is reframed from one of sequential parameter tuning to one of **constraint-aware, multivariable design-space reasoning**. This reframing underpins the development of biofabrication methodologies that emphasize reproducibility, robustness, and scalability over isolated performance optimization.

To consolidate the key coupling relationships and associated challenges discussed in this subsection, Table 2 summarizes the principal links between fabrication stages and biological considerations, highlighting the role of formal design frameworks in organizing these interactions.

## 4. Optimization and Control Strategy

Once biofabrication processes are represented within a structured mathematical framework, the natural next step is to formulate bioprinting as a constrained optimization and control problem. Rather than selecting fabrication parameters heuristically, predictive bioprinting requires objective functions that quantify desired outcomes; constraints that reflect physical, biological, and manufacturing limits; and strategies for navigating the resulting trade-offs. In this section, optimization and control concepts are introduced as the mathematical mechanisms that translate process–structure–function representations into actionable design decisions. By formalizing objectives, constraints, and sensitivity considerations, the proposed framework establishes a quantitative basis for reproducible, robust, and clinically relevant biofabrication workflows.

### 4.1. Objective Functions in Predictive Bioprinting

Within a predictive biofabrication framework, the selection of fabrication parameters is formulated as an optimization problem in which desired outcomes are expressed through explicitly defined objective functions. These objective functions translate qualitative design goals—such as construct fidelity or biological safety—into quantitative criteria that can be evaluated, compared, and optimized within the mathematical design space introduced in Section 3.1. This formalization represents a fundamental departure from empirical tuning, enabling systematic exploration of competing design priorities.

The multi-objective formulation introduced in this framework is intentionally algorithm-agnostic. Depending on the application context, objectives may be combined using weighted-sum approaches for interpretability and early-stage design exploration or treated using Pareto-based evolutionary algorithms such as NSGA-II when complex trade-offs between structural fidelity, biological safety, and manufacturing efficiency must be resolved. The selection of a specific optimization algorithm is therefore considered application- and system-dependent rather than intrinsic to the framework itself.

One primary objective in extrusion-based bioprinting is the maximization of shape fidelity, which reflects the degree to which the printed construct reproduces the intended geometry [28,29,30]. Shape fidelity can be represented through a function $J_{f}\left(p,m\right)$ that quantifies deviations between the target scaffold architecture and the realized filament geometry, accounting for effects such as spreading, sagging, or dimensional inaccuracies induced by process conditions and material behavior. This objective captures the structural reliability of the fabrication process and is central to applications requiring geometric precision.

A second critical objective concerns the minimization of cell damage during fabrication [31,32]. From a modeling perspective, this objective can be expressed as a function $J_{v}\left(p,m\right)$ that penalizes excessive shear stress exposure, prolonged residence times, or unfavorable thermal conditions. While biological response is inherently complex, such objective functions provide a quantitative proxy for cellular safety by incorporating threshold-based or weighted measures linked to known viability constraints. Importantly, this formulation does not claim direct prediction of biological outcomes but rather establishes a principled means of incorporating biological considerations into the optimization process.

Manufacturing efficiency introduces an additional objective, often expressed through print time or throughput [33]. A function $J_{t}\left(p\right)$ may be defined to represent fabrication duration, reflecting practical constraints related to scalability and operational feasibility. In many cases, improving efficiency conflicts with objectives related to resolution or biological protection, underscoring the inherently multi-objective nature of bioprinting optimization.

Collectively, these objectives define a vector-valued optimization problem of the form:

$$\underset{p}{\min\;}J\left(p,m\right)=\left\{J_{f},J_{v},J_{t}\right\}$$

where no single objective can be optimized independently without influencing the others. This formulation highlights the need for multi-objective optimization strategies capable of identifying balanced solutions rather than absolute optima. By expressing fabrication goals in this manner, the framework enables transparent evaluation of trade-offs and provides a foundation for control strategies discussed in subsequent sections.

To summarize the principal objectives commonly encountered in predictive bioprinting and their methodological roles, Table 3 provides an overview of representative objective functions and their associated design implications.

### 4.2. Constraints and Trade-Offs

The optimization of 3D bioprinting processes is inherently constrained by a combination of physical, biological, and manufacturing limits that restrict the feasible design space. Unlike purely geometric or mechanical fabrication problems, biofabrication must satisfy multiple, often competing, constraints simultaneously [34]. These constraints are not secondary considerations but fundamental elements that shape both achievable performance and system reliability [35].

Mechanical constraints arise from the material response and structural requirements of the printed construct. Excessive deformation, filament collapse, or insufficient stiffness can compromise shape fidelity and mechanical integrity [36]. Such limitations impose upper and lower bounds on parameters such as extrusion pressure, printing speed, and crosslinking rates. From a mathematical perspective, these bounds define admissible regions within the process parameter space, beyond which stable fabrication cannot be guaranteed.

Biological constraints further restrict this space by imposing viability and functionality thresholds. Cells embedded within bioinks are sensitive to shear stress, temperature variations, and exposure duration during fabrication. Exceeding critical thresholds can lead to reduced viability or altered biological behavior, even if structural objectives are met [37]. These constraints introduce non-negotiable limits that cannot be relaxed without undermining the biological purpose of the construct, reinforcing the need for explicit constraint formulation within the optimization framework.

Manufacturing constraints add an additional layer of complexity. Practical considerations such as printer resolution, operational stability, material availability, and production time impose limits that are independent of biological or mechanical performance [38]. For example, parameter combinations that yield optimal biological outcomes may be impractical at scale due to excessive print duration or sensitivity to minor perturbations. These constraints highlight the gap between laboratory optimization and translational feasibility.

The coexistence of mechanical, biological, and manufacturing constraints makes trade-offs unavoidable. Improvements in one objective often degrade another, as illustrated by the tension between printing speed and resolution, or between mechanical stability and nutrient transport. Within the proposed framework, these trade-offs are not treated as obstacles but as structured relationships that can be explored quantitatively. By defining constraints explicitly and embedding them into the optimization problem, the framework enables the identification of balanced solutions that respect all critical limitations rather than privileging a single performance metric.

**Figure 2:**
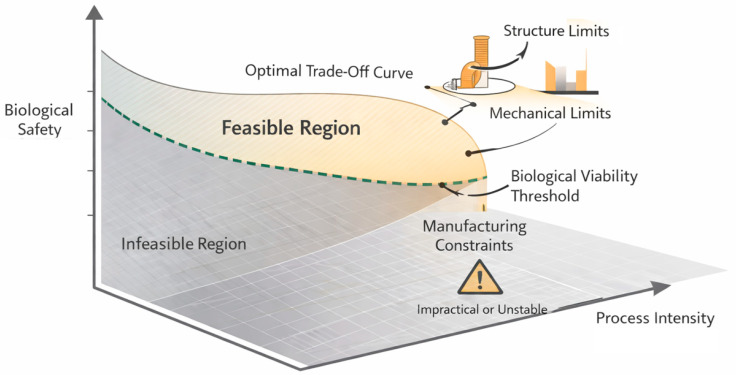
Conceptual illustration of constrained optimization in 3D bioprinting, highlighting the feasible design region defined by mechanical, biological, and manufacturing constraints and the resulting trade-offs between competing objectives.

To illustrate how competing objectives and constraints interact within the biofabrication design space, a schematic representation of the constrained optimization landscape is provided in Figure 2.

### 4.3. Sensitivity, Robustness, and Reproducibility

Optimized bioprinting parameters are only meaningful if their performance remains stable under realistic sources of variability. In practice, biofabrication processes are subject to fluctuations arising from material heterogeneity, biological variability, and machine-specific tolerances [39]. Even modest deviations in extrusion conditions or material response can alter filament formation, transport behavior, or cellular exposure, with downstream effects on construct quality [40]. For this reason, predictive bioprinting frameworks must account not only for optimal solutions but also for their sensitivity to perturbations.

Sensitivity analysis provides a structured means of examining how changes in individual process or material variables influence key performance indicators. Within the proposed framework, such analyses allow the relative influence of different parameters to be assessed without exhaustive experimentation [41]. Parameters associated with steep response gradients can be identified as critical control variables, while those exhibiting limited influence may be deprioritized in both modeling and experimental design. This distinction is particularly valuable in biofabrication contexts, where the dimensionality of the parameter space often exceeds what can be explored empirically.

Beyond local sensitivity, robustness considerations address the broader question of whether acceptable performance can be maintained across a range of operating conditions [42]. Rather than targeting narrowly defined optima, robust design strategies favor parameter regions that tolerate uncertainty while remaining within mechanical, biological, and manufacturing constraints. In biofabrication, where biological systems inherently resist precise control, this emphasis on stability over optimality is especially relevant. Robust parameter regions are more likely to support consistent outcomes when workflows are transferred between platforms or scaled beyond laboratory prototypes [43].

These aspects are closely tied to reproducibility and standardization [44]. Fabrication protocols derived from sensitivity-aware and robustness-oriented analyses are less dependent on operator expertise and less vulnerable to uncontrolled variation. As a result, they provide a stronger foundation for protocol harmonization and cross-laboratory reproducibility [45]. From a translational perspective, this reliability is a prerequisite for regulatory acceptance, where predictable behavior and documented process windows are often more critical than peak performance metrics.

By embedding sensitivity and robustness considerations directly into the optimization framework, applied mathematical methods contribute to an alteration in biofabrication practice—from empirically tuned, system-specific workflows toward quantitatively defined and reproducible design spaces. This shift supports the long-term maturation of 3D bioprinting technologies and aligns biofabrication methodologies with established principles of engineered system development.

To consolidate the role of sensitivity and robustness analysis within predictive bioprinting, Table 4 summarizes key methodological elements and their relevance to reproducibility and translational readiness.

From a practical perspective, the primary insight provided by sensitivity and robustness analysis within the proposed framework is the identification of dominant versus secondary process variables in extrusion-based bioprinting. Parameters associated with steep constraint boundaries, such as extrusion pressure and printing speed, define narrow biologically admissible regions and therefore require tighter control. In contrast, parameters exhibiting weaker sensitivity may be adjusted to improve efficiency or flexibility without compromising biological feasibility. This distinction shifts parameter selection away from fine-tuned optimal points toward robust operating regions, which is essential for reproducibility across printers, materials, and experimental conditions.

## 5. Demonstrative Computational Case Study

To illustrate the practical applicability of the proposed mathematical framework, a simplified computational case study is considered. The purpose of this case study is not to reproduce a specific experimental setup or to validate biological performance, but to demonstrate how process parameters, material properties, and biological constraints can be systematically integrated within a unified modeling and optimization workflow. By focusing on a representative bioprinting scenario and employing parameter ranges reported in the literature, the case study provides a transparent and reproducible example of how the framework can be used to explore design trade-offs and inform parameter selection.

The case study is deliberately constructed at a conceptual level, with clearly stated assumptions and simplified relationships that preserve generality while avoiding system-specific claims. This approach allows the mathematical structure of the framework to be examined independently of experimental variability and highlights its role as a methodological tool rather than a predictive substitute for empirical investigation. In this context, the case study serves as an illustrative bridge between the theoretical formulation presented in previous sections and the broader implications for predictive and reproducible biofabrication workflows.

### 5.1. Case Study Description

The demonstrative case study is intentionally formulated without numerical instantiation, as its purpose is to illustrate the structure of the proposed framework and the resulting qualitative design logic, rather than to reproduce data for a specific material, printer, or biological system. Thus, this work is based on a simplified extrusion-based 3D bioprinting scenario representative of commonly employed biofabrication workflows. The scenario is intentionally generic and does not correspond to a specific printer, bioink formulation, or biological system. Its purpose is to illustrate how the proposed mathematical framework can be applied to structure parameter selection, optimization, and trade-off analysis in a transparent and reproducible manner.

The fabrication process is assumed to involve a single bioink deposited through a cylindrical nozzle under steady extrusion conditions. The controllable process variables are extrusion pressure $p_{e}$, printing speed $v_{p}$, and nozzle diameter $d_{n}$, while temperature effects are assumed to be regulated within a narrow range and are therefore treated as constant for the purposes of this illustration. Material behavior is characterized by an effective viscosity parameter η\etaη, representing the dominant rheological response during extrusion, and a simplified crosslinking rate parameter $k_{c}$, accounting for post-deposition solidification effects.

Parameter ranges are selected based on values commonly reported in the bioprinting literature, without targeting any specific material system. Extrusion pressure and printing speed are assumed to vary within ranges that ensure continuous filament formation, while nozzle diameter is chosen to reflect typical resolutions used in hydrogel-based bioprinting. The material parameters are treated as bounded quantities consistent with shear-thinning bioinks, acknowledging that their precise values may vary across formulations and experimental conditions.

Biological considerations are incorporated through proxy constraints rather than explicit biological modeling. Cell viability is represented indirectly by limiting shear stress exposure during extrusion, and nutrient transport considerations are reflected through architectural features such as filament spacing and porosity. No assumptions are made regarding specific cell types, proliferation rates, or tissue functions, and no claims are made regarding biological outcomes beyond these abstracted constraints.

Several simplifying assumptions are adopted to maintain clarity and generality. Flow conditions are assumed to be quasi-steady, thermal effects are neglected, and interactions between neighboring filaments are treated implicitly through geometric constraints rather than detailed mechanical contact models. These assumptions are not intended to reflect all aspects of real biofabrication processes but to isolate the mathematical structure of the framework and demonstrate its application without unnecessary complexity.

It is emphasized that this case study is demonstrative and intended solely to illustrate the proposed framework. It does not constitute experimental validation, nor does it aim to predict quantitative biological performance. Instead, it provides a controlled setting in which the relationships between process parameters, constraints, and optimization objectives can be examined, setting the stage for the mathematical implementation presented in the following subsection.

### 5.2. Mathematical Implementation

Building on the defined case study scenario, the demonstrative implementation formalizes the bioprinting process as a constrained optimization problem over the controllable process variables. The objective is not to compute specific numerical solutions, but to illustrate how the proposed framework organizes relationships between parameters, constraints, and performance criteria in a mathematically consistent manner.

The controllable design vector is defined as

$$p=\left\{p_{e},u_{p},d_{n}\right\},$$

where extrusion pressure $p_{e}$, printing speed $u_{p}$, and nozzle diameter $d_{n}$ are treated as independent decision variables within bounded domains derived from literature-consistent ranges. Material properties, represented by effective viscosity n and crosslinking parameter $k_{c}$, are treated as fixed inputs for the purpose of this illustration, while acknowledging that they may vary across bioink formulations.

A simplified relationship between process parameters and shear stress exposure is introduced to capture biological safety considerations. Shear stress τ during extrusion is modeled conceptually as a function of pressure, flow velocity, and nozzle geometry:

$$\tau =f_{\tau }\;\left(p_{e},u_{p},d_{n}\right),$$

where the specific functional form is not prescribed but assumed to be monotonic with respect to pressure and velocity and inversely related to nozzle diameter. This abstraction is sufficient to introduce viability-related constraints without invoking detailed rheological models.

Shape fidelity is represented through a structural deviation function $J_{f}\left(p\right)$, which penalizes departures from target filament dimensions and spacing. This function aggregates geometric inaccuracies arising from excessive spreading, insufficient material deposition, or instability during extrusion. Manufacturing efficiency is incorporated through a print time proxy $J_{t}\left(p\right)$, inversely related to printing speed and directly related to construct size.

The resulting multi-objective optimization problem is expressed as

$$\underset{p}{\min\;}J\left(p\right)=\left\{J_{f}\left(p\right),J_{t}\left(p\right)\right\},$$

subject to biological and mechanical constraints of the following form:

$$\tau \left(p\right)\leq \tau_{max},\;g_{m}\left(p,n,k_{c}\right)\leq 0,$$

where $\tau_{max}$ denotes an admissible shear stress threshold and $g_{m}$ represents aggregated mechanical or process stability constraints ensuring continuous and reliable extrusion.

Rather than identifying a single optimal solution, the formulation admits a set of feasible parameter combinations that balance competing objectives while respecting all constraints. In this context, solution approaches may include weighted objective aggregation, Pareto front identification, or constraint-based filtering of the design space. The choice of approach is not fixed in this demonstrative implementation, as the emphasis lies on illustrating the structure of the optimization problem rather than prescribing a specific algorithm.

This mathematical implementation highlights how biological considerations, manufacturing efficiency, and structural fidelity can be incorporated within a unified optimization framework. By making parameter dependencies and constraints explicit, the framework enables systematic exploration of design trade-offs and provides a foundation for robust, model-informed parameter selection, as discussed in the following subsection.

### 5.3. Model-Based Predictive Trends and Constraint-Driven Insights

The trends discussed in this subsection arise from the mathematical structure of the proposed framework rather than from experimental measurement. They are intended to illustrate how predictive reasoning emerges through constraint interaction, parameter coupling, and objective trade-offs, and should not be interpreted as quantitative biological predictions.

Although the demonstrative case study is intentionally simplified, the mathematical formulation reveals several qualitative trends that are consistent with established bioprinting intuition while making their interdependencies explicit. These trends do not represent experimental observations, but rather logical consequences of the defined relationships between process variables, constraints, and optimization objectives within the proposed framework.

One prominent trend concerns the influence of printing speed and extrusion pressure on competing performance criteria. Increases in printing speed tend to reduce fabrication time, improving manufacturing efficiency, but are simultaneously associated with elevated shear stress exposure and reduced deposition accuracy under fixed nozzle geometry. Conversely, lower speeds and pressures favor structural stability and biological safety but incur penalties in throughput. The framework makes these opposing tendencies explicit, highlighting the absence of a universally optimal parameter set and reinforcing the need for trade-off-aware design strategies.

Nozzle diameter emerges as a moderating parameter that reshapes these trade-offs rather than eliminating them. Larger diameters reduce shear stress sensitivity and expand the biologically admissible region of the design space, but at the expense of resolution and geometric precision. Smaller diameters enable finer architectural control while narrowing the feasible operating window due to increased sensitivity to flow conditions. These interactions illustrate how individual parameters cannot be assessed in isolation, as their effects are strongly conditioned by the broader process context.

The formulation also underscores the role of constraint boundaries in defining practical design regions. Biological viability thresholds and mechanical stability limits effectively carve out feasible subsets of the parameter space, within which optimization objectives can be meaningfully pursued. Rather than acting as secondary restrictions, these constraints shape the structure of admissible solutions and guide the identification of robust operating regions that tolerate variability without violating critical limits.

From a design perspective, the primary insight offered by the demonstrative case study is the value of reframing parameter selection as a constrained, multivariable problem. Even in the absence of system-specific calibration, the framework provides a structured lens through which competing objectives and constraints can be examined simultaneously. This perspective supports informed decision-making and reduces reliance on sequential empirical adjustments, particularly when transitioning between materials, platforms, or application scales.

Taken together, these framework-implied trends illustrate how applied mathematical formulations can organize complex biofabrication considerations into interpretable design logic. While quantitative predictions require system-specific modeling and experimental integration, the qualitative insights derived here demonstrate the framework’s capacity to support predictive reasoning and robust parameter selection in bioprinting workflows. The qualitative trends and associated design implications emerging from the demonstrative case study are synthesized in Table 5.

### 5.4. Retrospective Consistency with Published Extrusion Bioprinting Studies

To further examine the predictive reasoning capability of the proposed mathematical framework, a retrospective consistency analysis was conducted using representative extrusion-based bioprinting studies reported in the literature. Several published works have systematically investigated the influence of extrusion pressure, printing speed, and nozzle geometry on filament stability and cell viability under controlled experimental conditions [25,31,36,38,39]. Although these studies were not originally designed to validate mathematical models, they provide well-documented parameter regimes associated with both biologically admissible and non-admissible outcomes.

When the process parameters reported in these studies are mapped onto the design space defined in Section 3.1, the proposed framework consistently identifies regions associated with elevated shear exposure and reduced biological admissibility. In particular, combinations of high extrusion pressure and elevated printing speed, which have been experimentally linked to decreased cell viability and increased cellular damage [25,31,36], fall outside the biologically constrained feasible regions defined by the framework. Conversely, parameter regimes reported to support high viability and stable extrusion behavior [38,39] lie within admissible regions bounded by shear stress and process stability constraints.

Importantly, this correspondence emerges without fitting model parameters to experimental data or introducing system-specific calibration. The framework does not seek to reproduce absolute viability values or to predict quantitative biological outcomes. Instead, it anticipates the structure of feasible and infeasible process regions based on the interaction between process variables, material response, and biological constraints. In this sense, prediction is expressed through the exclusion of non-viable operating regimes and the identification of constraint-consistent parameter domains prior to fabrication.

This retrospective consistency does not constitute experimental validation. Rather, it demonstrates that the proposed framework supports predictive reasoning at a methodological level by reproducing experimentally observed trends through first-principles structure rather than empirical tuning. Such capability is particularly relevant for early-stage design, protocol transfer, and scalability assessment, where anticipating admissible process windows is often more critical than numerical prediction of biological performance.

## 6. Discussion

The framework developed in this work is intended to function as a methodological contribution rather than a system-specific solution. Its value therefore lies less in the numerical outcomes of the demonstrative case study and more in the way it restructures how biofabrication problems are posed and examined. In this section, the implications of adopting a mathematically grounded approach to 3D bioprinting are discussed with respect to emerging scaffold design strategies, challenges associated with scale and translation, and the inherent limitations of abstraction-based modeling. By explicitly setting the proposed framework within these broader contexts, the discussion aims to clarify both its potential utility and the conditions under which it should be applied with caution.

### 6.1. Implications for Smart Scaffolds and Advanced Biofabrication

Recent developments in tissue engineering increasingly favor scaffold architectures that exhibit spatial variation rather than uniformity [46]. Gradients in porosity, stiffness, or material composition are often introduced to better replicate biological heterogeneity or to guide cell behavior across a construct [47]. While such designs are conceptually appealing, their practical realization through bioprinting remains challenging, as small changes in fabrication parameters can lead to pronounced and sometimes unintended structural variations [48]. Within this context, the mathematical framework proposed in this study offers a means of treating gradient features as deliberate design choices governed by quantifiable relationships, rather than as emergent effects that must be corrected empirically.

The relevance of predictive modeling becomes even more pronounced when scaffold designs are required to adapt across different regions of a construct or across successive fabrication stages. Adaptive biofabrication strategies—such as varying deposition conditions in response to local geometric or material requirements—benefit from formulations that explicitly relate process inputs to structural outcomes [49]. By expressing these relationships within a defined design space, parameter adjustments can be evaluated in advance, reducing reliance on iterative tuning and enabling more controlled transitions between design regimes.

**Figure 3:**
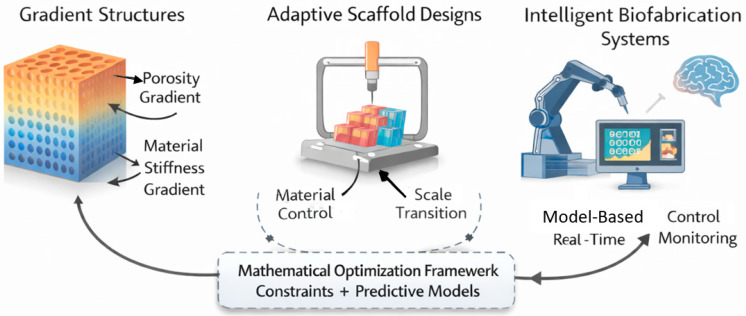
Conceptual illustration of how mathematically structured design frameworks support gradient scaffold architectures, adaptive biofabrication workflows, and the development of intelligent bioprinting systems.

At the system level, the same mathematical structure supports the conceptual development of intelligent biofabrication platforms. In such platforms, fabrication decisions are informed by model-based reasoning rather than solely by operator experience. Although the present work does not implement real-time feedback or closed-loop control, the framework is compatible with such extensions. Its primary contribution in this regard is the formalization of dependencies that must be understood before intelligent control strategies can be meaningfully deployed. In this sense, applied mathematical methodologies act as an enabling substrate for advanced biofabrication systems, rather than as an added layer of computational complexity. The role of mathematically structured design in enabling advanced biofabrication strategies, including gradient scaffolds, adaptive fabrication workflows, and intelligent system concepts, is schematically illustrated in Figure 3

### 6.2. Scalability, Reproducibility, and Translational Deployment in Biofabrication

Scalability represents one of the most persistent challenges in the transition of bioprinting technologies from laboratory demonstrations to clinically and industrially relevant applications [50]. Fabrication protocols that perform reliably at small scales or under tightly controlled experimental conditions often exhibit reduced robustness when extended to larger constructs, higher production volumes, or alternative hardware platforms [51]. In many cases, this loss of reliability can be traced to the absence of predictive models capable of accounting for how process parameters interact across scales and under variable operating conditions [52].

Within this context, mathematically grounded frameworks provide a practical mechanism for anticipating scale-dependent effects rather than reacting to them empirically. By formalizing relationships between process inputs, material behavior, and performance constraints, predictive models allow parameter ranges to be assessed for stability before fabrication is attempted. This capability becomes increasingly important as construct dimensions grow, since transport limitations, mechanical integrity, and process-induced stresses tend to intensify with scale. Rather than relying on direct extrapolation from small-scale experiments, model-informed approaches offer a structured basis for adjusting fabrication strategies in a manner that remains consistent with biological and manufacturing constraints.

Predictive modeling also plays a critical role in addressing reproducibility, which is a central concern in regulatory and clinical contexts [53]. From a translational perspective, the ability to define admissible process windows and to document their sensitivity to variability is often more valuable than achieving marginal gains in performance. Regulatory frameworks typically prioritize consistency, traceability, and risk mitigation, all of which benefit from design methodologies that explicitly identify critical parameters and acceptable tolerances. In this sense, applied mathematical tools support not only optimization but also compliance with emerging expectations for process standardization in biofabrication.

It is important to note that predictive frameworks do not eliminate the need for experimental validation or biological assessment [54]. Instead, they provide a means of structuring experimental effort by narrowing the range of plausible operating conditions and clarifying where uncertainty is most consequential. When integrated with empirical testing, such frameworks can reduce developmental overhead and support more efficient iteration toward clinically viable solutions. As bioprinting continues to move toward regulated applications, the role of mathematically informed design is therefore likely to shift from an optional enhancement to a foundational component of scalable and translatable biofabrication workflows.

### 6.3. Limitations of the Proposed Framework

While the proposed framework provides a structured and generalizable approach to predictive bioprinting, it is necessarily subject to limitations that arise from both modeling abstraction and the inherent complexity of biological systems. These limitations do not undermine the methodological contribution of the work, but they define the boundaries within which the framework should be interpreted and applied.

Importantly, the absence of experimental fitting in the present study is intentional, as it allows the framework to be evaluated on its structural predictive capability rather than on system-specific calibration. From a practical perspective, the framework is actionable in that it defines admissible parameter regions, objective trade-offs, and constraint interactions that can be directly used to guide experimental design, simulation studies, or process monitoring strategies.

A primary limitation concerns the absence of experimental validation within the present study. The framework and demonstrative case study are intended to illustrate methodological structure rather than to produce quantitative predictions for a specific biofabrication system. As such, the relationships between process parameters, material behavior, and biological performance are expressed at a conceptual or phenomenological level. Experimental data remain essential for calibrating model parameters, refining assumptions, and assessing biological outcomes in application-specific contexts. The framework should therefore be viewed as complementary to empirical investigation rather than as a substitute for it.

Modeling assumptions represent an additional source of limitation. To maintain clarity and generality, several simplifications were adopted, including steady-state flow conditions, reduced representations of material rheology, and proxy-based treatment of biological constraints. These assumptions are common in early-stage methodological work, but they inevitably restrict the resolution at which complex phenomena such as non-linear viscoelastic behavior, transient flow effects, or heterogeneous cellular responses can be captured. Extending the framework to incorporate more detailed physics or biology will require additional model development and system-specific data.

While finite element or computational fluid dynamics simulations could be coupled with the proposed framework to quantitatively resolve filament morphology, porosity, and local flow fields, such implementations are intentionally beyond the scope of the present methodological study. The aim here is to establish a general mathematical structure capable of organizing process–structure–function relationships across bioprinting systems, rather than to provide system-specific numerical predictions. Nevertheless, the formulation readily supports integration with CFD or FEM solvers as a downstream module, enabling detailed geometric and transport analysis once material models, boundary conditions, and validation data are specified.

Finally, the framework abstracts biological complexity into a limited set of performance metrics and constraints. While this abstraction is necessary for mathematical tractability, it cannot fully represent the adaptive, multiscale, and context-dependent nature of living systems. Cellular behavior, tissue maturation, and long-term functionality are influenced by factors that extend beyond fabrication parameters alone, including biochemical signaling, dynamic remodeling, and host interactions. These aspects lie outside the immediate scope of the present work but must be considered when translating predictive design frameworks into biological or clinical practice.

Recognizing these limitations is essential for responsible application of the proposed methodology. Rather than offering definitive solutions, the framework provides a structured starting point for integrating mathematical reasoning into biofabrication workflows. Its value lies in organizing complexity, clarifying trade-offs, and guiding experimental and computational efforts toward more reproducible and scalable outcomes.

## 7. Conclusions and Future Work

This work has presented a generalized applied mathematical framework for predictive 3D bioprinting, aimed at structuring the complex relationships between fabrication parameters, material behavior, scaffold architecture, and biological performance. Rather than advancing a system-specific model or reporting experimental outcomes, the contribution of this study lies in formalizing biofabrication as a constrained, multivariable design problem. By doing so, it offers a methodological foundation that supports quantitative reasoning, trade-off analysis, and reproducible decision-making in bioprinting workflows.

The demonstrative computational case study illustrates how such a framework can be used to organize parameter selection and optimization logic without relying on empirical calibration or platform-dependent assumptions. Through symbolic formulation and qualitative trend analysis, the framework highlights the limitations of isolated parameter tuning and underscores the value of integrated representations that explicitly account for biological and manufacturing constraints. In this sense, the work contributes to the development of biomimetic biofabrication methodologies by shifting emphasis from trial-and-error experimentation toward structured, model-informed design.

Several directions for future work naturally follow from this methodological foundation. Integration with experimental studies represents a critical next step, enabling model parameters to be calibrated, assumptions to be refined, and biological performance metrics to be evaluated in application-specific contexts. Such integration would allow the framework to evolve from a general design tool into a predictive instrument tailored to particular materials, cell types, and fabrication platforms.

The framework also provides a natural point of connection to emerging digital representations of biofabrication processes. When mathematical descriptions are coupled with experimental measurements acquired during fabrication, it becomes possible to construct process-aware digital counterparts that reflect both intended design and realized outcomes. In situ monitoring techniques, including imaging- and sensor-based approaches, could supply the data needed to update such representations as fabrication progresses, enabling deviations to be identified and addressed at an early stage. Although these capabilities are not implemented here, the structure of the proposed framework is compatible with their future integration.

In parallel, the explicit formulation of objectives, constraints, and design variables creates opportunities for the selective use of data-driven methods. Machine learning and related techniques may assist in navigating complex parameter spaces or in identifying regions of stable operation, particularly when experimental data become available. Their effectiveness, however, depends on being anchored to physically and biologically meaningful representations. In this respect, mathematically defined frameworks offer a necessary point of reference, ensuring that computational acceleration does not come at the expense of interpretability or control.

Taken together, these considerations point to a broader role for applied mathematics in the evolution of 3D bioprinting. By framing biofabrication as a structured design problem rather than a sequence of empirical adjustments, mathematical methodologies support more transparent, reproducible, and scalable workflows. The framework presented in this study is intended as a step in that direction, providing a common conceptual basis upon which experimental, computational, and biological efforts can be more effectively aligned.

## Figures and Tables

**Table 1:** Characteristics and limitations of prevailing biofabrication paradigms compared with the requirements for predictive and scalable 3D bioprinting workflows.

Aspect	Predominant Biofabrication Paradigms	Requirements for Predictive Biofabrication
Parameter selection	Empirical, trial-and-error tuning	Model-driven, quantitatively defined
Treatment of process variables	Adjusted individually or sequentially	Coupled, multivariable representation
Optimization strategy	Local, system-specific	Global, constraint-aware optimization
Reproducibility	Highly sensitive to setup and operator	Robust to parameter variation
Scalability	Limited transferability across scales	Explicitly considered through modeling
Biological performance prediction	Qualitative or post hoc	Integrated into design objectives
Use of computational tools	Fragmented, isolated simulations	Unified mathematical framework
Decision-making	Experience-based	Quantitative and predictive

**Table 2:** Key process–structure–function couplings in 3D bioprinting and corresponding methodological challenges addressed by applied mathematical frameworks.

Coupling Stage	Primary Factors	Biological Implications	Methodological Challenge
Process → Structure	Extrusion pressure, printing speed, nozzle diameter, rheology	Filament geometry, porosity, mechanical integrity	Nonlinear, multivariable dependence
Structure → Transport	Pore size, connectivity, scaffold architecture	Nutrient diffusion, waste removal	Spatial heterogeneity and scale effects
Process → Cell Exposure	Flow conditions, shear stress during extrusion	Cell viability, damage thresholds	Defining safe operational envelopes
Structure → Mechanics	Material stiffness, crosslinking kinetics	Cell mechanotransduction, tissue stability	Time-dependent property evolution
Structure → Function	Architecture-driven microenvironment	Tissue maturation and remodeling	Coupling short-term fabrication with long-term outcomes
Integrated System	Process, material, and biological constraints	Overall construct performance	Managing trade-offs and competing objectives

**Table 3:** Representative objective functions in predictive 3D bioprinting and their roles within the optimization framework.

Objective Category	Representative Objective	Design Focus	Methodological Role
Structural fidelity	Maximize shape fidelity	Geometric accuracy and reproducibility	Quantifies deviation from target architecture
Biological safety	Minimize cell damage	Viability and shear protection	Incorporates biological constraints into optimization
Manufacturing efficiency	Minimize print time	Scalability and throughput	Balances performance with practical feasibility
Resolution control	Balance resolution vs. speed	Feature accuracy	Manages trade-offs between detail and efficiency
Integrated performance	Multi-objective formulation	Overall construct quality	Enables trade-off-aware decision-making

**Table 4:** Sensitivity and robustness considerations in predictive 3D bioprinting and their implications for reproducibility and translation.

Aspect	Focus	Methodological Role	Practical Relevance
Sensitivity analysis	Parameter perturbation effects	Identifies dominant process variables	Prioritizes control and monitoring
Stable design regions	Low response variability	Supports robust parameter selection	Enhances repeatability
Robust optimization	Performance under uncertainty	Favors resilient solutions	Reduces failure risk
Variability sources	Material and system heterogeneity	Explicitly acknowledged in modeling	Improves transferability
Reproducibility	Consistent fabrication outcomes	Model-informed protocol definition	Facilitates clinical and regulatory adoption

**Table 5:** Qualitative trends and design implications identified through the demonstrative computational case study.

Aspect	Observed Trend	Design Implication
Printing speed	Higher speeds improve efficiency but increase shear exposure	Requires balancing throughput with biological constraints
Extrusion pressure	Elevated pressure enhances flow stability but narrows viability margins	Must be bounded by biological thresholds
Nozzle diameter	Larger diameters expand feasible regions but reduce resolution	Acts as a trade-off moderator rather than an optimizer
Constraint interaction	Biological and mechanical limits define admissible regions	Feasible design space is shaped by constraints, not objectives alone
Parameter coupling	Variables exhibit interdependent effects	Isolated tuning is insufficient for robust design
Optimization outcome	No single global optimum exists	Multi-objective and robustness-aware strategies are required

## Data Availability

Data is contained within the article.
